# Effect of Surface Inhomogeneity of Ion-Exchange Membranes on the Mass Transfer Efficiency in Pulsed Electric Field Modes

**DOI:** 10.3390/membranes10030040

**Published:** 2020-03-11

**Authors:** Dmitrii Butylskii, Ilya Moroz, Kseniya Tsygurina, Semyon Mareev

**Affiliations:** Membrane Institute, Kuban State University, 149 Stavropolskaya st., 350040 Krasnodar, Russia; ilya_moroz@mail.ru (I.M.); kseniya_alx@mail.ru (K.T.); mareev-semyon@bk.ru (S.M.)

**Keywords:** electrodialysis, ion-exchange membrane, pulsed electric field mode, voltammetry, electroconvection, increasing of mass transfer, visualization

## Abstract

Despite the growing interest in pulsed electric field modes in membrane separation processes, there are currently not many works devoted to studying the effect of the surface properties and composition of ion-exchange membranes on their efficiency in these modes. In this paper, we have shown the effect of increasing mass transfer using different kinds of ion-exchange membranes (heterogeneous and homogeneous with smooth, undulated, and rough surfaces) during electrodialysis in the pulsed electric field modes at underlimiting and overlimiting currents. It was found that the maximum increment in the average current is achieved when the average potential corresponds to the right-hand edge of the limiting current plateau of the voltammetric curve, i.e., at the maximum resistance of the system in the DC mode. For the first time, the development of electroconvective vortices was visualized in pulsed electric field modes and it was experimentally shown that even at relatively low frequencies, a non-uniform concentration field is preserved at the time of a pause, which stimulates the rapid development of electroconvection when pulses are switched on again. In the case of relatively high pulse frequencies, the electroconvective vortices formed during a pulse lapse do not completely decay during a pause; they only slightly decrease in size.

## 1. Introduction

In recent years, in the field of electrodialysis, considerable interest has been focused on the study of intense current regimes. The use of such regimes allows one to save on the most expensive component of the system—the ion-exchange membrane [1,2]. The operation of the electrodialysis apparatus in these modes is accompanied by the development of coupled effects of concentration polarization, such as electro- and gravitational convection, and the water splitting at the membrane/solution interface [3,4,5,6,7].

One of the main mechanisms of overlimiting mass transfer is electroconvection, which not only increases the effective transport of salt ions [8] but also partially suppresses the water splitting at the interface [9], which impedes the process of precipitation in the electrodialyzer chambers [10].

It is known that the greatest influence on the development of electroconvection is exerted by the electric and/or geometric heterogeneities of the membrane surface [11,12,13,14,15], hydrophobicity of the surface and its charge [16,17], and heterogeneity of the concentration distribution at the membrane/solution interface [3]. All these effects are synergistic and contribute to the appearance of the tangential component of the electric force, which sets the volume of the solution at the membrane surface in motion [2,18].

As it was shown in the theoretical works of Davidson at al. [13] and Rubinstein and Zaltzman [4], to intensify electroconvective mixing, the length of the elementary region on the surface of a heterogeneous membrane (the sum of the lengths of the conductive and non-conductive regions) should correspond to the channel width of the electrodialyzer. In the case of a flow electromembrane system, the size of electroconvective vortices will be limited by the thickness of the diffusion layer [19].

The presence of a geometric heterogeneity in the form of waviness or other types of relief on the membrane surface, as well as electrical heterogeneity, leads to a significant increase in the mass transfer rate [12,20,21,22] due to several effects: Increase in the active area of the membrane available for mass transfer; increase in the contribution of forced convection due to improved hydrodynamic conditions, and increase in the tangential component of electric force [20,23].

Another effective way to intensify electroconvection is to use pulsed electric field (PEF) modes [24,25,26,27]. The essence of the electrodialysis desalination of solutions using PEF modes is to alternate cycles of switching on and off the external electric field. It allows the reduction in concentration polarization and, as a result, increase in mass transfer rate [24,27,28], reduction in the rate of water dissociation at the membrane/depleted solution boundary [29], as well as the rate of precipitate formation in the electrodialyzer chambers during the processing of multicomponent solutions [30,31,32]. Nevertheless, energy consumption in the PEF mode should be greater [28].

At the underlimiting current regimes of the PEF, the above-mentioned effects are achieved due to partial relaxation of the concentration profile near the membrane surface, and the solution resistance is significantly reduced. Therefore, a high current value is reached at the beginning of the next cycle [28]. On average, the increment in mass transfer rate per cycle is due to the high contribution of electromigration transport when the voltage is switched on again. A similar effect is observed with metal deposition on the electrode [33]. The upper limit of the mass transfer rate is reached when the thickness of the solution layer, in which changes in the concentration occur during the application of pulses, becomes sufficiently small [28]. Thus, the mass transfer rate in the PEF mode increases with increasing frequency and decreasing duty cycle. According to [28], in electrodialysis, this dependence was first established by Karlin and Kropotov [34,35].

When using the PEF modes at overlimiting currents during a pause (where the current is zero), the concentration field does not disappear [25,36]. Its non-uniformity plays the same role as the membrane surface electrical heterogeneity by favoring the formation of new electroconvective vortices [3]. The results of the calculation [25] showed that the reason for the prolonged lifetime of the vortices after switching off the current/voltage is the non-uniform distribution of the electric body force at the membrane surface.

Currently, the PEF modes are actively used in electrodialysis for processing complex solutions of the food industry [31,37,38,39]. However, the effect of the surface properties of ion-exchange membranes on the process efficiency has not been studied enough. It was shown in [10] that the hydrophobicity and heterogeneity of the ion-exchange membrane surface lead to the development of electroconvection. This results in an increase in the rate of demineralization at PEF modes. On the other hand, heterogeneity of the ion-exchange membrane leads to the blocking of pores by the precipitates, turning into non-conductive regions, thus leading to the fast increase in resistance observed from the beginning of the current pulses [40].

Electrochemical methods (such as voltammetry, chronopotentiometry, and electrochemical impedance spectroscopy) are usually used to study the coupled effects of concentration polarization and, in particular, electroconvection [5,8,11,12]. These methods allow important quantitative characteristics that determine the economic indicators of the process to be obtained [2]. However, for the elucidation of occurring phenomena mechanisms, the received information is indirect.

The development of experimental methods for visualizing electroconvection and other related effects of concentration polarization is a necessary step to deepen the knowledge in this field of science and, in particular, for verifying assumptions and developing mathematical models. In 2008, a joint team of scientists from Ben-Gurion University of the Negev (Israel) and University of Twente (The Netherlands) proposed an effective way to directly visualize electroconvective vortices [41] by adding 1 μm neutrally buoyant polystyrene tracer particles to the electrolyte solution. It should be noted that the phenomenon of electroconvection began to be considered as generally recognized in the literature only after the publication of the visualization results. Later, similar visualization methods were developed in other leading research centers [41,42,43,44,45].

It is worth noting that other methods for the visualization of electroconvection were previously known. The scientific group of Shaposhnik and Vasil’eva used the laser interferometry method to visualize concentration profiles at the surface of ion-exchange membranes. These concentration profiles change their form due to the development of electroconvection [46,47]. The Schlieren-diagonal method also allows one to monitor changes in concentration at the membrane surface [48].

The methods for visualization make it possible to determine, for example, the moment of vortices occurrence at the membrane surface in the electro-membrane system with an increase in the set current [45]; the effect of the alternating current frequency on the size of the vortices [49]; the influence of the shape of the surface geometric heterogeneity of the ion-conductive material on the size of the vortices [50].

In this work, we evaluated the effect of the surface properties of ion-exchange membranes on the efficiency of mass transfer using PEF modes. Additionally, the kinetics of electroconvective vortices formation near the surface of an ion-exchange membrane in these electric field modes is visualized for the first time.

## 2. Materials and Methods

### 2.1. Membranes

Homogeneous (Nafion 438 and Neosepta AMX) and heterogeneous (M1, M2, MK-40) membranes, which differ in surface structure and chemical composition, were chosen as the objects of study.

**Figure 1:**
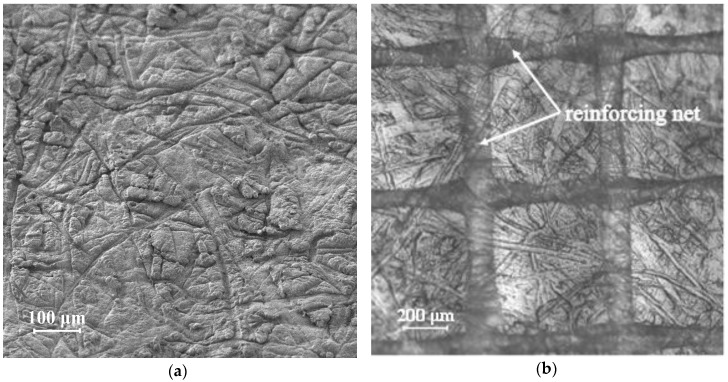
SEM image of the surface of a homogeneous Nafion 438 membrane (**a**) and its micrograph obtained using an optical microscope (**b**).

Nafion 438 (DuPont, Wilmington, DE, USA) is a homogeneous perfluorosulfonic cation-exchange membrane. A distinctive feature of this membrane is a flat profile on one side and a rough and undulated surface on the other side (Figure 1a). In addition, the membrane is reinforced with a polytetrafluoroethylene net (Figure 1b).

**Figure 2:**
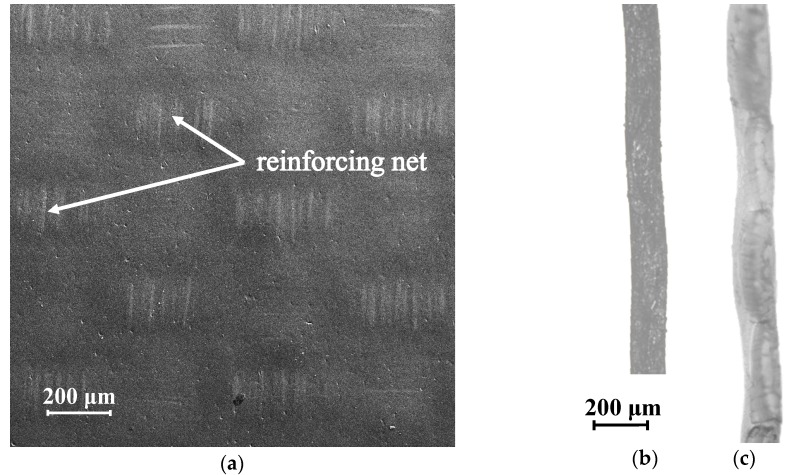
SEM image of the surface of a homogeneous Neosepta AMX membrane (**a**) and micrograph of its cross-sections in a dry (**b**) and swollen state (**c**) (b and c were adapted from [51]).

The homogeneous Neosepta AMX anion-exchange membrane (Astom Corp., Tokyo, Japan) consists of a copolymer of styrene-divinylbenzene (45–65%) with fixed quaternary amino groups and polyvinyl chloride (45–55%). The membrane is reinforced with a polyvinyl chloride mesh, which causes the undulation of its surface. It is visualized in the form of longitudinal strips in the vicinity of the surface (Figure 2a). The wave height varies from 10 μm in the dry state (Figure 2b) to 30 μm in the swollen state (Figure 2c) [51].

The surface images of the membranes presented in Figure 1a and Figure 2a were obtained using a MERLIN scanning electron microscope (SEM) (Carl Zeiss, Jena, Germany). The images of dry (Figure 2b) and swollen membranes (Figure 1b and Figure 2c) were obtained using an optical microscope SOPTOP CX40M (Ningbo Sunny Instruments Co., Ltd., Yuyao, China).

**Figure 3:**
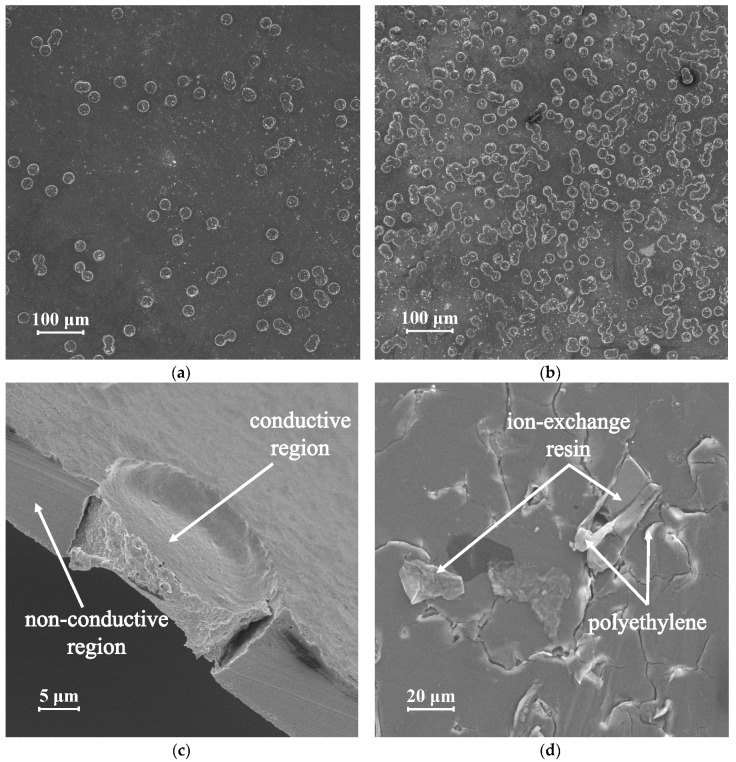
SEM images of the surface of the heterogeneous M1 (**a**) and M2 (**b**) membranes, as well as a cross-section through the conductive region of the membrane M1 (**c**) and the surface of the MK-40 (**d**) membrane.

The substrates (track-etched membranes) used in the fabrication of M1 (Figure 3a) and M2 (Figure 3b) membranes were produced from a 30 µm thick polyethylene terephthalate (PET) film (Hostaphan RNK, Mitsubishi Polyester Films) at JINR, Dubna. To make the substrates permselective, they were covered from one side with a dispersion of Nafion^®^ sulfonated perfluorinated material in alcohol; the Nafion^®^ material filled the pores (Figure 3c) and formed a ~3 µm thick layer on one side of the membrane. The second side of the membrane was heterogeneous: Only the pores filled with Nafion were conductive. Membranes differed in distribution density of the conductive regions on the heterogeneous surface and their diameter: For M1 and M2, the density of the conductive regions was 1.47 × 10^4^ and 8.78 × 10^4^ per square centimeter, respectively, and their average diameter was 25.9 and 21.5 μm, respectively; thus, the mean surface fraction of conductive regions on the heterogeneous side of the resulting ion-exchange membrane (IEM) was 7.7% for M1 and 31.8% for M2.

The densities of pore distribution and pore diameter were determined from the micrographs (Figure 3a,b) obtained using a Hitachi TM3000 scanning electron microscope (Hitachi High-Technologies Corp., Tokyo, Japan).

MK-40 (Shchekinoazot, Pervomayskiy, Tula Region, Russia) is produced by hot pressing the ion-exchange material (KU-2-8 cation-exchange resin), dispersed to linear dimensions of 5–50 μm, with high-density polyethylene as the inert binder. Most of the surface (about 85%) of the MK-40 membrane is covered with polyethylene film. The areas where grains of ion-exchanger come out of the surface are evenly distributed over the membrane surface (Figure 3d). The surface image of the MK-40 membrane (Figure 3d) was obtained using a MERLIN scanning electron microscope.

### 2.2. Experimental Setups and Solutions

Tests of the studied membranes in the PEF and DC modes, as well as measurement of the current–voltage curves (CVC), were carried out using the laboratory flow-through electrodialysis cell described in [52]. The experimental setup was described in detail in our previous work [27]. All measurements were conducted in 0.02 M NaCl solution. The linear flow velocity of the electrolyte solution was 3.6 mm/s.

To visualize the electroconvective vortices, a laboratory flow-through electrodialysis cell was used. Two Neosepta AMX anion-exchange membranes formed the studied channel of which the width of the chamber was 3.0 mm and the length was 5.0 mm. A mixture of 0.02 M NaCl and 10 μM Rhodamine 6G was used as a working solution for visualizing electroconvective flows and salt concentration. Visualization was carried out under conditions of laminar flow of an electrolyte solution with a linear velocity of 0.6 mm/s. Video recording of the formation of electroconvective vortices was carried out using an optical microscope SOPTOP CX40M (Ningbo Sunny Instruments Co., Ltd., Yuyao, China) equipped with a Toupcam camera (ToupTek Photonics Co., Ltd., Hangzhou, China).

### 2.3. PEF Protocol

**Figure 4:**
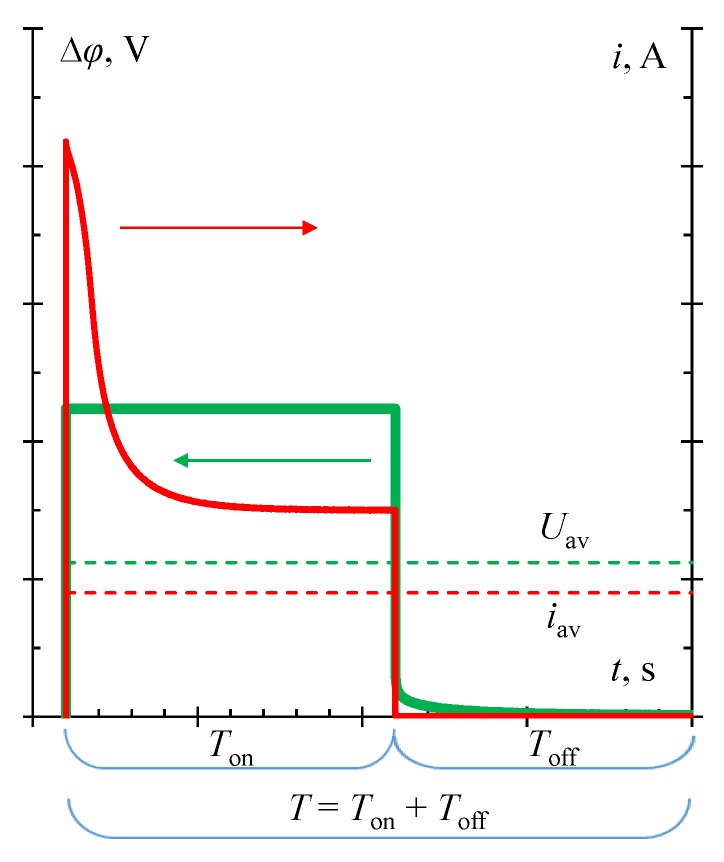
Schematic current and voltage versus time dependencies in pulsed electric field mode. The dashed lines show the period-averaged applied potential (*U*_av_) and the period-averaged measured current (*i*_av_).

The pulsed electric field experiment conditions were chosen as follows: 15 different frequencies (*f*) in a range from 0.01 to 20 Hz were used. A pulse lapse of constant voltage (*T*_on_) was alternated with the pause lapse of zero current (*T*_off_) (Figure 4).

We used five duty cycles (*α*) (1/4, 1/3, 1/2, 2/3, 3/4), where *α* is the ratio of *T*_on_ to (*T*_on_ + *T*_off_) (Figure 4). The voltages (*U*_av_) needed for the membrane systems to reach several selected current densities are found from current–voltage curves. We compared the systems at *i*/*i*_im_^Lev^ ratios 0.75, 1.0, 1.25, and 1.4, where *i*_lim_^Lev^ is the theoretical value of the limiting current calculated by the Leveque equation.

For example, we determined from a preliminarily obtained voltammetry curve of a hypothetical ion-exchange membrane that at a ratio *i*/*i*_im_^Lev^ = 1.0, the potential value is 0.5 V. This potential value in the PEF mode was used as *U*_av_, and in the DC mode, it was set as constant in time (*U*_DC_). The potential value at the pulse lapse of a constant voltage (*U*_on_) in the PEF mode is determined by the expression *U*_on_ = *U*_av_/*α*, and, for example, at α = 1/2, it will be equal to 1.0 V. At the time of a pause in the PEF mode, the current value is reset independently of the frequency and duty cycles; therefore, the potential at this time slowly decreases to zero or, if the pulse rate is high enough, reaches some minimum value (Figure 4). The average value for the period (*T*) of the measured current (*i*_av_) is determined from its time dependence presented in Figure 4 by the following expression:

(1) $$i_{\mathrm{av}}\left(A\right)=\frac{1}{T}\int_{0}^{T}idt,\;\mathrm{where}\;T\;=\;T_{\mathrm{on}}\;+\;T_{\mathrm{off}}.$$

When visualizing electroconvective vortices, a pulse lapse of a constant current (*T*_on_) is alternated with the pause lapse of zero current (*T*_off_). We used two frequencies (0.5 and 5.0 Hz) and one duty cycle value equal to 1/2. The average current for the period was set equal to 3.0 *i*_lim_^Lev^, where *i*_lim_^Lev^ was 0.55 mA.

## 3. Results and Discussion

### 3.1. The Study of Mass Transfer Rate

**Figure 5:**
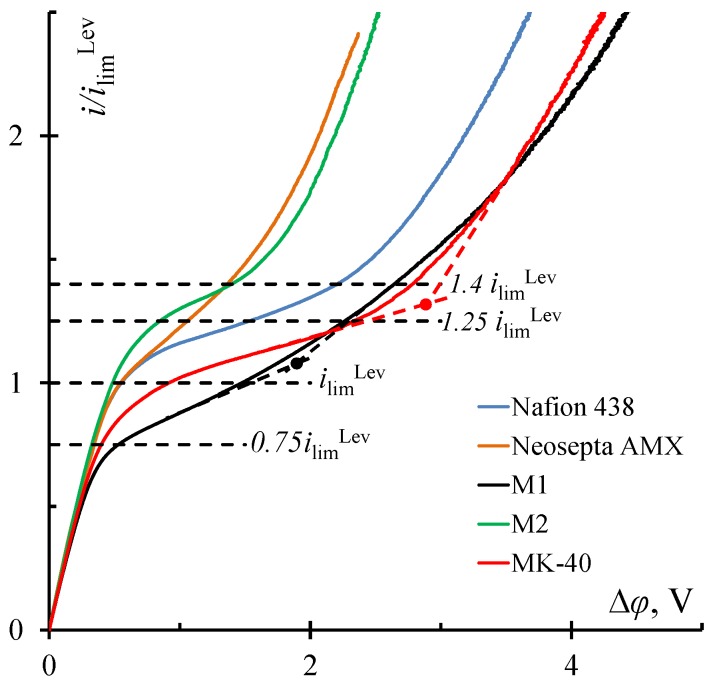
Current–voltage curves of the studied membranes in a 0.02 M NaCl solution at a linear flow velocity of 3.6 mm/s. Tangents and their intersection points show the right-hand edge of the limiting current plateau for M1 and MK-40 membranes.

Current–voltage curves were obtained to determine the effect of the surface structure and chemical composition of the studied ion-exchange membranes on their electrochemical characteristics (Figure 5). The limiting current for homogeneous membranes Nafion 438 and Neosepta AMX, determined experimentally, exceeds the theoretical value calculated by the Leveque equation (*i*_lim_^Lev^ equals to 7.9 mA and 12.0 mA for cation- and anion-exchange membranes, respectively) and is shown in Figure 5 by a horizontal dashed line with the corresponding mark. The reason for this effect is the presence of a geometric heterogeneity in the form of a rough and undulated surface facing the desalination chamber of the electrodialysis cell, in the case of the Nafion 438 membrane (Figure 1a), and a wavy surface, in the case of the Neosepta AMX membrane (Figure 2c).

In the case of heterogeneous membranes, the limiting current and the plateau length are mainly a function of the ratio of conductive/non-conductive regions [15]. It is known from the literature that the fraction of the conductive surface of a commercial MK-40 membrane can reach up to 30% and substantially depends on the state of the membrane (dry/swollen) [53]. Particles of the ion-exchange material emerging on the surface are randomly distributed and have different shapes, and their size varies in the range from 4 to 50 μm [52,53,54]. A small fraction of the conductive surface causes a low value of limiting current for a given membrane (Figure 5).

Membranes M1 and M2 are identical but differ in the fraction of conductive regions that are easily visualized (Figure 3a,b). Earlier, we tested these samples and showed the influence of the fraction of the conductive region on various electrochemical characteristics of the membranes [55,56,57]. It was found that a low fraction of conductive regions on the surface of the M1 membrane leads to a low value of the limiting current (Figure 5). Apparently, the fraction of conductive regions in the case of the M2 membrane is large enough for the development of electroconvection, which is the reason for the high limiting current and the short plateau.

**Figure 6:**
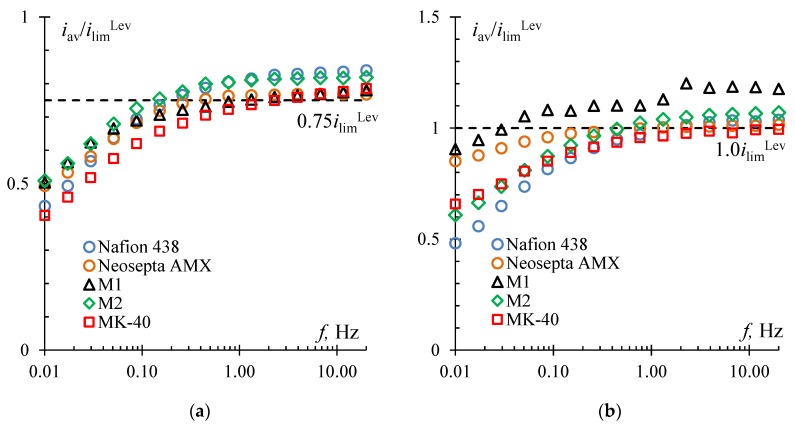
The results of testing the Nafion 438, Neosepta AMX, M1, M2, and MK-40 membranes in the pulsed electric field mode: *i* = 0.75 *i*_lim_^Lev^, duty cycle 1/4 (**a**), and 1.0 *i*_lim_^Lev^, duty cycle 1/4 (excluding results for Neosepta AMX membrane for which they are shown at 1.0 *i*_lim_^Lev^ and *α* = 2/3) (**b**). The horizontal dashed line denotes the *i*/*i*_lim_^Lev^ ratio corresponding to a given potential drop *U*_av_ in the DC mode.

In Figure 5, dashed lines indicate the currents at which the studied membrane samples were compared in the PEF mode. It was found that if the average switching potential in the PEF mode corresponds to underlimiting currents, it is possible to obtain only a small increase in the average current over a period (Figure 6a,b), which, under the condition of a low water splitting rate [29], characterizes the increase in mass transfer rate in the PEF mode as compared to the DC mode where the same potential drop is set.

The results obtained are in good agreement with the data presented in [27,28]. With an increase in the pulse rate and a decrease in the duty cycle, the mass transfer rate increases. For all studied membrane samples with an average potential drop corresponding to 0.75 *i*_lim_^Lev^, the highest average current value for the period was reached at a duty cycle of 1/4 (Figure 6a). The long pause was necessary for the concentration of counterions at the membrane surface to take the value as close as possible to the concentration in the bulk solution. It explains the efficiency of the regime in which the current is set to zero in most of the period (*T*_off_ = 3/4*T* at *α* = 1/4). At the beginning of the next cycle, when setting a large potential drop (*U*_on_ = 4*U*_av_ at *α* = 1/4), after a pause, the maximum value of the average current over the period among all duty cycles was reached. This is because the resistance became lower during the pause at *α* = 1/4 than with any other value of the duty cycle from those studied in this work.

It is worth noting that the largest increase in the average current value was obtained for Nafion 438 and M2 membranes, which have good current–voltage curves. This was apparently due to more favorable conditions (surface roughness and undulation, and a high fraction of conductive regions, respectively, as well as high hydrophobicity of the surface in both cases) for the development of electroconvective vortices at the pulse lapse of a constant voltage (*U*_on_) equal to *U*_av_ /*α* = 0.35 × 4 V = 1.4 V in the case of Nafion 438 and M2 membranes compared to other samples, although *U*_av_ at 0.75 *i*_lim_^Lev^ for the other membranes was slightly larger (M1 and MK-40) or had the same value (Neosepta AMX) (Figure 5).

**Figure 7:**
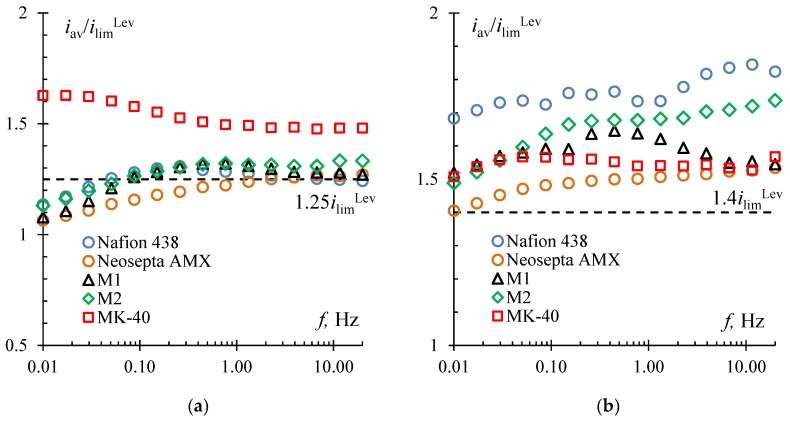
The results of testing the Nafion 438, Neosepta AMX, M1, M2, and MK-40 membranes in the pulsed electric field mode: *i* = 1.25 *i*_lim_^Lev^, duty cycle 1/2 (excluding results for M1, M2 membranes for which they are shown at 1.25 *i*_lim_^Lev^ and *α* = 1/3) (**a**), and 1.4 *i*_lim_^Lev^, duty cycle 1/2 (excluding results for Neosepta AMX membrane for which they are shown at 1.4 *i*_lim_^Lev^ and *α* = 3/4) (**b**). The horizontal dashed line denotes the *i*/*i*_lim_^Lev^ ratio corresponding to a given potential drop *U*_av_ in the DC mode.

Table 1 shows the values of the maximum average increment in measured current over the period, *ω* (%), in the PEF mode at the most effective duty cycle, *α*, (indicated in parentheses in Table 1) compared to the DC mode for the selected *i*/*i*_im_^Lev^ ratios. The obtained values of the increment correspond to the maximum points in the frequency dependences of the average current value presented in Figure 6a,b and Figure 7a,b. The formula determines the increment values, ω (%), (Table 1):

(2) $$\omega (\%)=(i_{\mathrm{av}}/i-1)\times 100\%$$

where *i*_av_ is the average value of the measured current in the PEF mode over the period, determined by Equation (1), A; *i* is the current value achieved in the DC mode when the potential drop *U*_DC_ corresponding to *U*_av_ in the PEF mode is set on the membrane under study, A.

With an average potential drop corresponding to 1.0 *i*_lim_^Lev^, the highest average current value for the period when using the PEF modes was also achieved with a duty cycle of 1/4 (Figure 6b, Table 1). The only exception is the Neosepta AMX membrane, for which at *i* = 1.0 *i*_lim_^Lev^, the most effective duty cycle was 2/3, but no significant increase in mass transfer rate was observed (only 2.0%) (Table 1).

However, it was found that in the case of using the M1 membrane in the PEF mode with a potential drop corresponding to 1.0 *i*_lim_^Lev^, the increase in mass transfer rate compared to the DC mode can be significant (Figure 6b) and equal to about 20% (Table 1).

A further increase in the set potential drop leads to significant energy consumptions both in the DC and in the PEF mode. For example, for the MK-40 membrane, with an increase in the potential drop from a value corresponding to 1.0 *i*_lim_^Lev^ to a value corresponding to 1.25 *i*_lim_^Lev^, it leads to an increase in energy consumption by almost six times (from 1.8 to 10.3 mW cm^−2^ per half-cell pair) in the case of the DC mode and by two times (from 7.3 to 15.4 mW cm^−2^ per half-cell pair) in the case of the PEF mode.

However, it was shown that with a potential drop corresponding to 1.25 *i*_lim_^Lev^, in the case of the MK-40 membrane, there is a significant increase in the mass transfer rate by almost 30% (Table 1) compared to the DC mode (Figure 7a). It should be noted that the frequencies at which the maximum mass transfer rate for a given membrane is observed become quite small (<0.1 Hz), and the optimal duty cycle for almost all membranes becomes 1/2.

The optimal value of the duty cycle equal to 1/2 at overlimiting currents was also determined in [31,37], including the case of using homogeneous Neosepta membranes in electrodialyzers [58,59]. Probably, the surface inhomogeneity of the MK-40 membrane leads to the shift in the optimal frequency to a relatively low-frequency range. As was shown in [25,36], a higher average current value is achieved due to the development of electroconvection. In the case of heterogeneous membranes, the size of electroconvective vortices depends on the length of the elementary unit of heterogeneity and may be larger than that near the surface of homogeneous membranes under similar conditions [13]. During a pause in the case of a homogeneous membrane, the transport of electrolyte ions is mainly due to diffusion from the bulk solution in the normal direction to the membrane surface. These ions are transported not only in the normal but also in the tangential direction along non-conductive sections in the case of a heterogeneous membrane [19]. As a result, more time is needed for efficient mass transfer in the case of a heterogeneous membrane, which leads to a shift in the optimal frequency to a relatively low-frequency range.

The same effect was also manifested on other heterogeneous membranes under study at a potential drop corresponding to 1.4 *i*_lim_^Lev^. For membranes M1 and M2, the optimal frequency value also shifted to the low-frequency range and was approximately equal to 0.5 Hz (Figure 7b).

For homogeneous Nafion 438 and Neosepta AMX membranes, a noticeable increase in the average current value was also observed at a potential drop corresponding to 1.4 *i*_lim_^Lev^. However, the course of the frequency dependencies for Nafion 438 and Neosepta AMX with the most optimal duty cycle equal to 1/2 and 3/4, respectively, remained the same as in the underlimiting current regimes. As in our previous work [27], we assumed that in this case, a certain resonance phenomenon occurs. The essence of this phenomenon is in the fact that the concentration field formed during a short pause effectively stimulates the increase in electroconvection after the application of a second voltage pulse.

It was found that the maximum increment in the average current is achieved when the average potentials correspond to the right-hand edge of the limiting current plateau in the voltammetric curves. For example, for the M1 membrane, the maximum increase was observed at a potential drop corresponding to 1.0 *i*_lim_^Lev^. However, its current–voltage curve (Figure 5) shows that the experimental value of the limiting current for this membrane is low. In the region of the potential drop, corresponding to 1.0 *i*_lim_^Lev^, an inflection was observed, i.e., the transition of the electromembrane system from the limiting state to the overlimiting one.

A similar transition and maximum increase in the average current were observed at 1.4 *i*_lim_^Lev^ for M2, Nafion 438, and Neosepta AMX membranes and at 1.25 *i*_lim_^Lev^ for the MK-40 membrane (Figure 5, Table 1).

**Figure 8:**
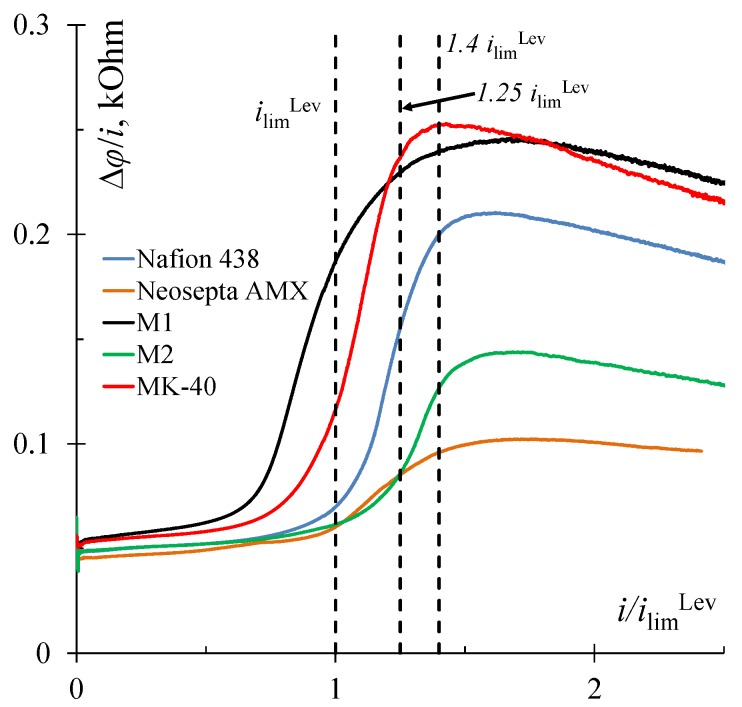
Resistance (∆*ϕ*/*i*) versus *i*/*i*_lim_^Lev^ dependencies for the studied membranes determined from current–voltage curves (Figure 5).

It should be noted that the maximum increment in the average current for all studied samples was also achieved when the average potential corresponded to 1.4 *i*_lim_^Lev^ (Table 1). The maximum resistance of the electro-membrane system was achieved at this point on the current–voltage curves (Figure 8). That is, this current was the least attractive for electrodialysis at the DC mode. When using the PEF mode, this resistance decreased markedly because, during pulses with a high switching potential and pauses with zero current, favorable conditions for the development of electroconvection were made at the membrane surface.

Thus, the surface properties of ion-exchange membranes affect the limiting current value and CVC plateau length (Figure 5), which ultimately affects the efficiency and energy consumption of electrodialysis desalination both in the PEF and DC modes. The maximum current increase in the PEF compared to the DC mode at underlimiting and overlimiting currents was achieved at α = 1/4, and at potentials corresponding to overlimiting currents, at α = 1/2 (Table 1). Apparently, this is because electroconvection is absent or develops weakly at the underlimiting and limiting currents. Therefore, the increment in the mass transfer rate in the PEF mode is associated with a significant decrease in the solution resistance during a pause, which should be long.

### 3.2. Visualization of Electroconvective Vortices

The formation of electroconvective vortices in a PEF mode was visualized near the surface of the Neosepta AMX membrane at frequencies of 0.5 and 5.0 Hz, which, as was established in the previous section, are included in the optimal frequency ranges for the maximum increase in mass transfer rate. The duty cycle value of 1/2 was chosen as the most characteristic optimal value for overlimiting current regimes.

**Figure 9:**
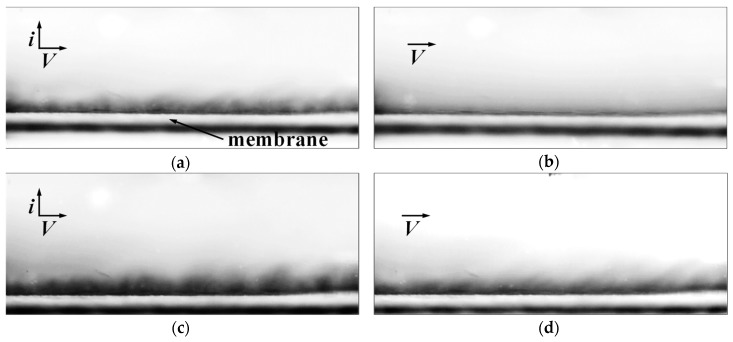
Visualization of the vortex structures at the Neosepta AMX membrane surface in the desalination channel of the electrodialysis cell in the DC mode at *i* = 3.0 *i*_lim_^Lev^ (**a**) and in the absence of current (**b**), and in the PEF mode at *i*_av_ = 3.0 *i*_lim_^Lev^ (*f* = 0.5 Hz, *α* = 1/2) at the pulse lapse of a direct current (**c**) and pause lapse of zero current (**d**).

As it can be seen from the videos (Supplementary Materials) and Figure 9, the profile of the membrane under study is visible at the bottom of the image. When setting the DC mode at the current corresponding to 3.0 *i*_lim_^Lev^ (Figure 9a), in the video recording (Video 1), it can be seen that the formation of electroconvective vortices after the current is turned on (the current turns on for about 5 s) takes about 15 s. Dark areas correspond to a low concentration of the solution, light ones to a high concentration. The shape of the electroconvective vortices is clearly visible. The average size of the vortices increases with time (Video 1).

When using the PEF mode at the same average current value (over the period) (*i*_av_) corresponding to 3.0 *i*_lim_^Lev^ and a pulse frequency of 0.5 Hz, electroconvective vortices formed much faster. This was because the set current was two times higher than the average one at the pulse lapse (*α* = 1/2) (Video 2). The next time the pulse lapse of a direct current switched on, the vortices reached the maximum possible size (Figure 9c). It can be seen that, at the time of the pause, the vortices had time to decay. However, the inhomogeneous concentration field was preserved and did not have time to completely wash off by the laminar flow of the electrolyte (Figure 9d). This heterogeneity stimulates the development of electroconvection [25] in the same way it occurs in the case of an electrically inhomogeneous surface, which was first shown in the theoretical work of Urtenov et al. [3].

In the case where a relatively high pulse frequency (5.0 Hz) was set, an increase in the size of vortices in time was also observed (Video 3). However, during a pause, the electroconvective vortices formed during the pulse lapse of a direct current did not completely decay, as was established in theoretical works [25,36]. During the pause, the electroconvective vortices had time only to decrease slightly in size. Alternating vortex sizes during the pulse lapse of a direct current and pause lapse of zero current are visually observed in Video 3 in the form of a flickering concentration field.

It was shown in [25] that after turning off the voltage in the membrane system, electroosmotic fluid flows are retained for some time. These flows are due to the uneven distribution of the concentration of counterions along the longitudinal coordinate inside the electric double layer. The consequence of this is the uneven distribution of the volumetric electric force that feeds the vortices.

## 4. Conclusions

In this paper, the effect of surface inhomogeneity of ion-exchange membranes on the mass transfer efficiency in PEF modes was studied. It was found that the maximum increment in the average current is achieved when the average potentials correspond to the right-hand edge of the limiting current plateau in the voltammetric curves. In addition, it was shown that the maximum current increase in the PEF mode compared to the DC mode at underlimiting and limiting currents is most often achieved at α = 1/4, and at potentials corresponding to overlimiting currents, at α = 1/2.

It has been established that at potentials corresponding to overlimiting currents, a shift in the optimal frequency in the PEF mode to a relatively low-frequency range is observed. It is assumed that this is due to the relaxation rate of the concentration field at the membrane surface during a pause, which is low in the case of heterogeneous membranes compared to homogeneous ones.

It was experimentally shown that at relatively low frequencies in the PEF mode at the time of a pause, the vortices have time to decay. However, the inhomogeneous concentration field is retained and does not have time to completely wash off by the laminar flow of the electrolyte. It stimulates the rapid development of electroconvection when the electric field is switched back on. In the case where a relatively high pulse frequency is set during a pause, the electroconvective vortices formed during the pulse lapse of a direct current do not completely decay; they only slightly decrease in size.

## Figures and Tables

**Table 1:** The values of the maximum average increment in measured current over the period, *ω* (%) *, at the most effective duty (the value of *α* is indicated in round brackets) in the pulsed electric field (PEF) mode compared to the DC mode.

Set Potential Drop (*U*_av_) at *i/i*_lim_^Lev^	Nafion 438	Neosepta AMX	M1	M2	MK-40
0.75	12.0 (*α* = 1/4)	4.0 (*α* = 1/4)	4.6 (*α* = 1/4)	9.2 (*α* = 1/4)	4.7 (*α* = 1/4)
1.0	3.9 (α = 1/4)	2.0 (*α* = 2/3)	20.1 (*α* = 1/4)	7.0 (*α* = 1/4)	0.2 (*α* = 1/4)
1.25	4.6 (*α* = 1/2)	1.6 (*α* = 1/2)	5.6 (*α* = 1/3)	6.6 (*α* = 1/3)	30.1 (*α* = 1/2)
1.4	32.9 (*α* = 1/2)	11.1 (*α* = 3/4)	19.3 (*α* = 1/2)	25.9 (*α* = 1/2)	19.2 (*α* = 1/2)

* average values from 3 experiments.

## Supplementary Materials

The following are available online at https://www.mdpi.com/2077-0375/10/3/40/s1, Video 1: Visualization of the vortex structures at the Neosepta AMX membrane surface in the desalination channel of the electrodialysis cell in the DC mode at *i* = 3.0 *i*_lim_^Lev^; Video 2: Visualization of the vortex structures at the Neosepta AMX membrane surface in the desalination channel of the electrodialysis cell in the PEF mode at *i*_av_ = 3.0 *i*_lim_^Lev^ (*f* = 0.5 Hz, *α* = 1/2); Video 3: Visualization of the vortex structures at the Neosepta AMX membrane surface in the desalination channel of the electrodialysis cell in the PEF mode at *i*_av_ = 3.0 *i*_lim_^Lev^ (*f* = 5.0 Hz, *α* = 1/2).
